# Unraveling NEAT1's complex role in lung cancer biology: a comprehensive review

**DOI:** 10.17179/excli2023-6553

**Published:** 2024-01-03

**Authors:** Md Sadique Hussain, Obaid Afzal, Gaurav Gupta, Ahsas Goyal, Waleed Hassan Almalki, Imran Kazmi, Sami I. Alzarea, Abdulmalik Saleh Alfawaz Altamimi, Neelam Kukreti, Amlan Chakraborty, Sachin Kumar Singh, Kamal Dua

**Affiliations:** 1 School of Pharmaceutical Sciences, Jaipur National University, Jagatpura 302017, Jaipur, India; 2 Department of Pharmaceutical Chemistry, College of Pharmacy, Prince Sattam Bin Abdulaziz University, Al Kharj, 11942, Saudi Arabia; 3 School of Pharmacy, Suresh Gyan Vihar University, Jagatpura 302017, Mahal Road, Jaipur, India; 4 Centre for Transdisciplinary Research, Saveetha Institute of Medical and Technical Science, Saveetha University, Chennai, India; 5 Institute of Pharmaceutical Research, GLA University, Mathura, U. P., India; 6 Department of Pharmacology, College of Pharmacy, Umm Al-Qura University, Makkah, Saudi Arabia; 7 Department of Biochemistry, Faculty of Science, King Abdulaziz University, Jeddah, Saudi Arabia; 8 Department of Pharmacology, College of Pharmacy, Jouf University, Sakaka, Al-Jouf, Saudi Arabia; 9 School of Pharmacy, Graphic Era Hill University, Dehradun 248007, India; 10 Faculty of Biology, Medicine and Health, The University of Manchester, Oxford Road, Manchester M13 9PL, U.K.; 11 Cardiovascular Disease Program, Biomedicine Discovery Institute and Department of Pharmacology, Monash University, Clayton, VIC 3800, Australia; 12 School of Pharmaceutical Sciences, Lovely Professional University, Phagwara, Punjab, 144411, India; 13 Faculty of Health, Australian Research Centre in Complementary and Integrative Medicine, University of Technology Sydney, Ultimo, Australia; 14 Discipline of Pharmacy, Graduate School of Health, University of Technology Sydney, NSW 2007, Australia

**Keywords:** long non-coding RNA, NEAT1, lung cancer, clinical applications, cancer

## Abstract

This review delves into the pivotal role of the long non-coding RNA NEAT1 in cancer biology, particularly in lung cancer (LC). It emphasizes NEAT1's unique subcellular localization and active involvement in gene regulation and chromatin remodeling. The review highlights NEAT1's impact on LC development and progression, including cell processes such as proliferation, migration, invasion, and resistance to therapy, positioning it as a potential diagnostic marker and therapeutic target. The complex web of NEAT1's regulatory interactions with proteins and microRNAs is explored, alongside challenges in targeting it therapeutically. The review concludes optimistically, suggesting future avenues for research and personalized LC therapies, shedding light on NEAT1's crucial role in LC.

See also the Graphical abstract(Fig. 1).

## Background

Among various types of cancer, lung cancer (LCs) is the most prevalent and is recognized as a significant contributor to tumor-related mortality worldwide. Within the realm of LCs, non-small-cell lung cancer (NSCLC), encompassing the predominant subtype, constitutes 70 % to 80 % of the overall cases (Zappa and Mousa, 2016[89]). LC's incidence surpasses 1 in 10 of all malignant tumor cases globally. Notably, the occurrence rate of LCs is higher than most other malignancies worldwide (Araghi et al., 2023[2]). As per the American Cancer Society's projections for 2023, it is anticipated that there will be about 1,958,310 new cases of cancer and approximately 609,820 cancer-related deaths in the United States. For patients diagnosed with NSCLC falling within stages I to III, it is encouraging that around 70 % of them can potentially achieve curative outcomes through surgical interventions. In stark contrast, survival rates beyond five years for individuals with advanced NSCLC are dishearteningly low, hovering at a mere 5 %. In the clinical landscape, the triad of chemotherapy, targeted therapy, and immunotherapy emerges as the three foremost treatment modalities employed in managing advanced cases of NSCLC. Still, the outlook stands unfavorable (Cascone et al., 2006[6]; Chen et al., 2023[9]; Ko et al., 2018[36]; Siegel et al., 2023[67]). As a result, present NSCLC therapy techniques must be improved.

NEAT1 (Nuclear Enriched Abundant Transcript 1) represents a prominent example of long non-coding RNAs (lncRNAs) that exhibit substantial expression in diverse cancer types, such as breast, gynecologic, lung, esophageal, colorectal, hepatocellular, and endometrial cancers (Dong et al., 2019[13]; Hussain et al., 2023[28]; Thankachan et al., 2021[72]; Yu et al., 2017[88]). This lncRNA plays a pivotal role as a structural constituent within paraspeckles (PSs), dynamic nuclear bodies devoid of membranes, which exert influence over various cellular processes, including stress responses (Hussain et al., 2023[27]; Taiana et al., 2020[71]). Intriguingly, NEAT1 has been implicated in intricate cellular functions encompassing cell cycle modulation, proliferation, apoptosis, migration, and DNA damage repair in tumorigenic contexts (Huang et al., 2020[25]; Nitusca et al., 2021[61]; Wu et al., 2019[78]). Furthermore, its involvement extends to tumor development and prognosis, where elevated NEAT1 levels correlate with unfavorable outcomes among cancer patients (Taiana et al., 2020[71]; Thankachan et al., 2021[72]; Zhang et al., 2022[94]). Elucidating its mechanistic underpinnings, NEAT1 operates as a microRNA (miRNA) sponge, facilitating the proliferation of tumor cells by sequestering miRNAs (Zhang et al., 2022[94]). Impressively, the diagnostic potential of NEAT1 emerges as a noteworthy biomarker, particularly promising for managing breast and gynecologic malignancies (Thankachan et al., 2021[72]). Beyond cancer, NEAT1's therapeutic relevance extends to conditions like neurodegenerative diseases and additional tumor types (Taiana et al., 2020[71]).

NEAT1 emerges as a notable lncRNA, exhibiting robust expression across diverse cancer types, including LCs (Jiang et al., 2021[31]; Nitusca et al., 2021[61]; Wu et al., 2019[78]). NEAT1 also serves as a promising indicator, showing potential for managing LCs (Nitusca et al., 2021[61]). Studies have unveiled NEAT1's upregulation in NSCLC cell lines compared to normal lung epithelial cells, wherein inhibition of NEAT1 yielded suppression of NSCLC progression (Wu et al., 2019[78]). Furthermore, NEAT1's involvement in promoting sarcoma metastasis via RNA splicing pathway regulation and its role in triggering a tumor-like phenotype in lung bronchial epithelial cells through HIF-1α activation following PM2.5 exposure have been elucidated (Huang et al., 2020[25], Jiang et al., 2021[31]). In a study involving mice carrying conditional mutations in Kras and Trp53 genes, NEAT1 exhibited significant upregulation in lung metastases, highlighting its association with metastatic processes. Clinically, co-overexpression of Oct4/NEAT1/MALAT1 emerged as an independent predictor of poor outcomes in LC patients (Huang et al., 2020[25]; Jen et al., 2017[30]; Kong et al., 2019[37]). Collectively, NEAT1 emerges as a pivotal player in LC biology, promising not only as a potential biomarker but also as a therapeutic target for LC management.

Research findings have consistently demonstrated that NEAT1 plays a role in the advancement of multiple cancer types, encompassing breast cancer, LCs, hepatocellular carcinoma (HCC), ovarian cancer, and prostate cancer (Fang et al., 2017[14]; Klec et al., 2019[34]; Liu et al., 2018[51]; Xiong et al., 2018[82]). Elevated NEAT1 expression frequently correlates with diminished survival rates among cancer patients (Luo et al., 2019[55]; Yu et al., 2020[86]). Furthermore, NEAT1 has been implicated in diminishing the responsiveness of cancers to chemotherapy or radiotherapy (Jiang et al., 2016[32]; Wang et al., 2020[73]). Nevertheless, an intriguing observation arises in acute myeloid leukaemia (AML), where NEAT1 overexpression appears to exert a safeguarding effect. Conversely, reduced NEAT1 levels have been associated with differentiation disorders in AML (Feng et al., 2020[16]; Zeng et al., 2014[90]). NEAT1 exhibits robust expression within LC tissues and cells (Chen et al., 2020[8]; Li et al., 2018[44]; Liu et al., 2020[49]; Ma et al., 2020[56]). The pivotal role of NEAT1 in LCs biology positions it as a promising biomarker and therapeutic target for effective LCs management (Chen et al., 2020[8]; Liu et al., 2020[49]). Notably, inhibiting NEAT1 activity has shown potential for suppressing the progression of NSCLC cells (Liu et al., 2020[49]; Zhao et al., 2014[98]). Its role extends to promoting tumor development, accompanied by an adverse prognostic implication among cancer patients (Zhao et al., 2014[98]). NEAT1 emerges as a pivotal participant in developing and progressing LCs, orchestrating various cellular functions. Its regulatory network encompasses diverse pathways, encompassing miRNA, epigenetic inhibition, and signaling cascades. Its multifaceted role of NEAT1 positions it as a promising therapeutic target for LC management.

## NEAT1 and its Functional Roles in Lung Cancer

### NEAT1 structure and localization 

NEAT1, a non-coding, polyadenylated transcript first identified in 2007, is abundant in the pancreas, prostate, ovary, and colon. It has been found to participate in multiple biological processes, encompassing the regulation of genes, the generation of miRNAs, and the cellular stress response. By modulating the functioning of genes linked to control of tumor cell proliferation, movement, spread, triggering of apoptosis, epithelial-to-mesenchymal transition (EMT), stem cell-like phenotype, resistance to chemo and radio-resistance, NEAT1 promotes the onset and growth of tumors. It exhibits typical traits of a tumor promoter because it initiates and advances tumors, and since it frequently shows instability in malignancies, which is correlated with clinical characteristics including metastasis, recurrences, and longevity of the individual (Dong et al., 2018[12]). The NEAT1 locus generates two transcripts, NEAT1.1 and NEAT1.2, used to make NEAT1. While NEAT1.1 (3.7 kb) is not necessary for creating paraspeckles, it is expected to serve a variety of paraspeckle-independent tasks. NEAT1.2 (23 kb) is the lengthier isoform required to build these nuclear entities. Due to their complementary base pairs, NEAT1.2 and NEAT1.1 have been projected to bind together at the 3′ end of NEAT1.2, although NEAT1.2 plays a more critical role in the creation of paraspeckles (Kukharsky et al., 2020[39]; Lin et al., 2018[47]; Sasaki et al., 2009[65]). While NEAT1.2 lacks a poly (A) end and is supported by a triple helical configuration at the 3′ ends, NEAT1.1 is maintained by a conventional poly (A) tail at the 3′ ends (Hirose et al., 2014[23]; Wang et al., 2020[76]). The structure of NEAT1.1 may be seen as a component of NEAT1.2, and these two variants are formed through different 3′-end sequencing (Naganuma and Hirose, 2013[59]). NEAT1.1 has strong expression in various organs, in contrast to the NEAT1.2 isoform, which was found in high quantities in certain types of cells in the intestines and stomachs of adult mice (Nakagawa et al., 2011[60]).

It appears that NEAT1.1 and NEAT1.2 play various operations in controlling the characteristics of cancerous cells. For instance, in colorectal cancer (CRC) cell lines, NEAT1.1 knockdown may lower cell spread and growth, but NEAT1.2 knockdown increases development. The same investigation revealed that liver metastatic lesions expressed NEAT1.1 substantially more than nearby healthy colorectal or primary CRC tissues (Wu et al., 2015[80]). These results showed that while NEAT1.2 may operate as a tumor inhibitor in CRC, NEAT1.1 could function as a carcinogenic agent. Different cell types seem to have different ways of controlling how NEAT1 variants are expressed (Nakagawa et al., 2011[60]).

NEAT1, an extensive lncRNA, demonstrates notable expression across various cancer types, including LCs. Its pivotal role within LC biology positions it as a potential biomarker and therapeutic target for effective LC management (Beeharry et al., 2018[4]; Benner and Mihailescu, 2020[5]; Gu et al., 2022[21]). In the context of LCs, NEAT1's predominant nuclear localization is notable. RNA probes recognizing both NEAT1 isoforms, specifically NEAT1-2, revealed NEAT1 foci in LC cells. These foci exhibited core-shell spheroidal structures, with NEAT1's 5' and 3' regions enveloping the central portion (Liu et al., 2022[48]).

### NEAT1 as an oncogenic lncRNA in lung cancer 

As indicated by a series of investigations (Li et al., 2019[41], 2018[44]; Zhang et al., 2017[92]), NEAT1, an oncogenic lncRNA, emerges as a significant contributor to the advancement of NSCLC. Elevated expression of NEAT1 is evident in NSCLC tissues, exhibiting strong correlations with advanced TNM stages, lymph node metastasis, distant metastasis, and unfavorable prognostic outcomes (Li et al., 2018[44]). *In vitro* experiments have substantiated the inhibitory impact of NEAT1 silencing on cell proliferation and invasion. The underlying mechanistic insights elucidate that NEAT1 exerts these effects by enhancing the expression of HMGB2, a gene targeted by miR-181a-5p, through competitive bidding, effectively acting as a "sponge" for miR-181a-5p. Furthermore, NEAT1 orchestrates resistance to paclitaxel in NSCLC by activating the Akt/mTOR signaling pathway (Li et al., 2019[41], 2018[44]). Remarkably, NEAT1 functions as a competing endogenous RNA (ceRNA) for miR-377-3p, counteracting its processes and consequently releasing the repression of endogenous targets, most notably E2F3, a central oncogene implicated in NSCLC progression (Fu et al., 2022[17]; Sun et al., 2016[69]; Zhang et al., 2017[92]). NEAT1's intricate roles in promoting NSCLC progression collectively underscore its oncogenic character and offer valuable insights into the mechanisms underpinning its contributions to this disease.

#### Interaction with molecular pathways involved in cell proliferation 

NEAT1 exhibits significant involvement in cellular proliferation across diverse cancer types. Particularly noteworthy is its role in HCC, where NEAT1 assumes a regulatory role in cell proliferation and the EMT. This regulation becomes pronounced as HepG2 cells advance towards the phenotypic characteristics associated with liver cancer. The expression of NEAT1 has been observed to correlate directly with unfavorable survival outcomes in multiple cancers, such as CRC and glioma. This lncRNA facilitates crucial growth, metastasis, and disease progression by activating the Wnt/β-catenin cascade (Xu et al., 2021[83]). In lung adenocarcinoma (LA), NEAT1 is a molecular sponge for miR-490-3p, impeding cancer by inhibiting the RhoA/ROCK cascade (Zhao et al., 2023[96]).

Similarly, in gastric carcinoma, NEAT1 orchestrates cellular behaviors encompassing proliferation, invasion, and apoptosis by modulating the miR-500a-3p/XBP-1 axis (Zhou et al., 2021[101]). The influence of NEAT1 extends to cholangiocarcinoma, where it drives cell proliferation, migration, and invasion through its interaction with the miR-186-5p/PTP4A1 axis (Li et al., 2021[43]). Furthermore, NEAT1 contributes to wound healing by enhancing the autophagy pathway via the NEAT1/miR-17-5p/Ulk1 axis (An et al., 2023[1]). Collectively, NEAT1 emerges as a pivotal player in the promotion of cell proliferation within diverse cancer categories. Its involvement is discerned through intricate molecular pathways that vary according to the specific cancer type under scrutiny.

#### Modulation of apoptosis and cell cycle regulation 

The expression of NEAT1 has demonstrated a significant association with unfavorable survival outcomes across a range of cancers. NEAT1 fosters growth, metastasis, and progression within these cancer types by activating the Wnt/β-catenin cascade, as established by existing research sources (Li et al., 2021[42]). Furthermore, NEAT1 has been identified as a promoter of wound healing, capitalizing on the autophagy pathway, which it enhances via the NEAT1/miR-17-5p/Ulk1 axis (Archambeau et al., 2019[3]). Despite these findings, our current understanding of NEAT1's involvement in apoptosis and cell cycle regulation remains limited. Nonetheless, investigations have indicated that NEAT1 assumes a role in suppressing apoptosis while promoting cell growth, migration, and invasion across diverse cancer categories (Li et al., 2021[42]). Nevertheless, comprehensive research efforts are necessary to gain a more profound comprehension of NEAT1's specific contributions to apoptosis and cell cycle regulation in the context of cancer. Ongoing studies are imperative to elucidate the full extent of NEAT1's role in these crucial cellular processes.

#### Promotion of metastasis and invasion

NEAT1 influences the advancement of LC cells by participating in the endogenous RNA network involving NEAT1/let-7a/IGF2 (Qi et al., 2018[62]). This regulatory role extends to modulating cell behaviors such as proliferation, invasion, migration, and apoptosis by targeting the has-miR-376b-3p/SULF1 axis within NSCLC (Chen et al., 2020[8]). Furthermore, NEAT1/miR-204/NUAK1 axis emerges as a promising therapeutic avenue for NSCLC (Zhao et al., 2020[97]). Within the context of LCs, the NEAT1/miR-1224/KLF3 axis influences cellular processes encompassing proliferation, apoptosis, and invasion (Yu et al., 2019[87]). Additionally, NEAT1 amplifies the trajectory of NSCLC by enhancing EIF4G2 via miR-582-5p sponging (Zhang et al., 2020[93]). A multi-faceted role is attributed to NEAT1, encompassing transcriptional activation of both NEAT1 via its promoter and MALAT1 via enhancer binding, thereby fostering cell proliferation, motility, and ultimately culminating in lung tumorigenesis and unfavorable prognosis (Jen et al., 2017[30]). Further augmenting this complexity, NEAT1 actively contributes to the progression of NSCLC by mediating the miR-101-3p/SOX9/Wnt/β-catenin signaling pathway (Kong et al., 2019[37]). Notably, NEAT1's involvement extends to promoting proliferation and invasion, an effect achieved via its interaction with miR-181a-5p in NSCLC (Li et al., 2018[44]). Figure 2(Fig. 2) (Reference in Figure 2: Li et al., 2018[44]) depicts the NSCLC cell migration and invasion.

## NEAT1's Regulatory Mechanisms

### NEAT1-miRNA Interactions

Many interconnected research endeavors have consistently showcased the pivotal involvement of NEAT1-miRNA interactions in the intricate process of tumorigenesis. These interactions encompass three primary forms: NEAT1 acting as a miRNA sponge, miRNAs subject to regulation by NEAT1, and NEAT1's regulatory influence by miRNAs.

#### NEAT1 is a sponge of miRNAs

**Figure 1:**
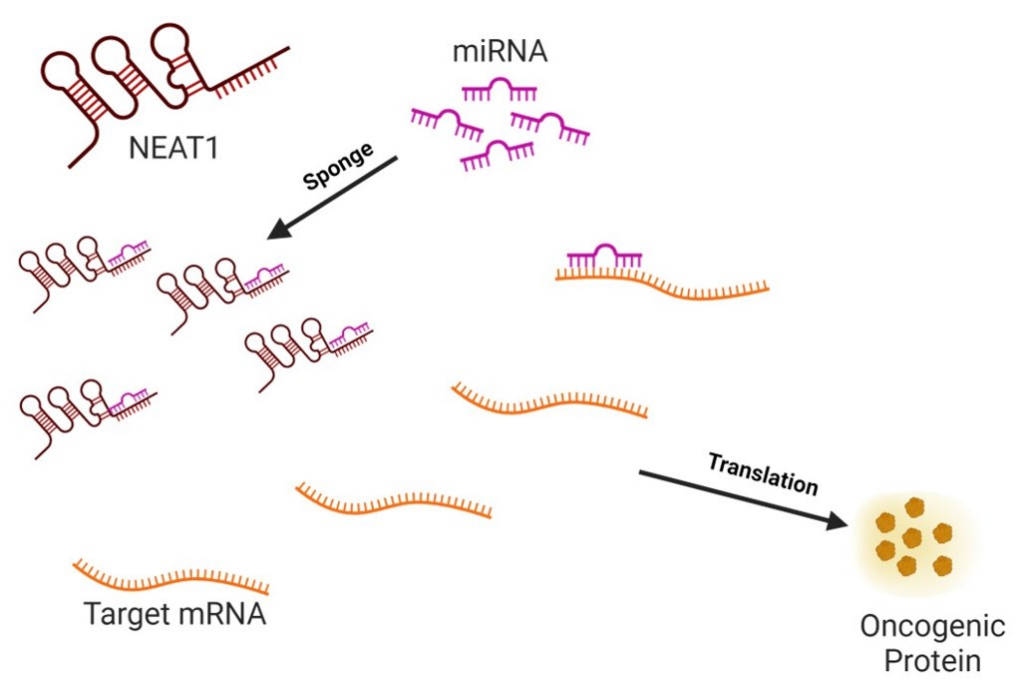
Graphical abstract

**Figure 2:**
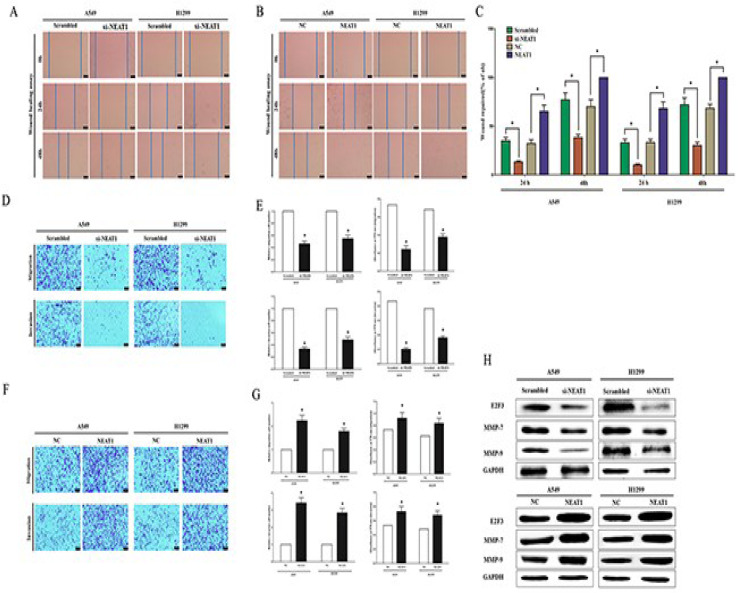
NEAT1 facilitates *in vitro* migration and invasion of NSCLC cells. The present study sought to investigate the impact of NEAT1 on the migration and invasion capabilities of NSCLC cells through a series of *in vitro* assays. (A-B) Representative photomicrographs are presented, depicting the "wound healing assay" progression in A549 and H1299 cells at 0 hours, twenty hours, and forty-eight hours post-transfection. The scale bar corresponds to 50 μm. (C) The outcomes of the statistical analysis conducted on the "wound healing assay" are illustrated. (D-G) A549 and H1299 cells were placed in the upper chamber of transwell inserts to evaluate cell migration and invasion. After twenty-four hours, cells that migrated to the lower chamber with the serum-supplemented medium were stained using 0.1 % crystal violet, visualized under a phase-contrast microscope, and documented in images. The scale bar signifies 50 μm. Manual cell counting was performed to determine the total cell count across five fields. (H) Additionally, the protein expression levels of E2F3, MMP-7, and MMP-9 were assessed in A549 and H1299 cells after transfection. All assays were performed in triplicate, and statistical significance was determined through the student's t-test. The data are presented as means ± SEM, with * indicating P < 0.05 (Li et al., 2018a).

ceRNA networks formed via lncRNA/ miRNA/mRNA interactions are integral to various cancer-related biological processes. NEAT1 has been observed to potentially compete with miRNAs for binding sites within the 3′-UTR of target mRNAs. This mode of interaction leads to the inhibition of miRNA function and subsequent activation of target proteins (depicted in Figure 1(Fig. 1), graphical abstract). As a notable molecular sponge, NEAT1 competes with various miRNAs to regulate oncogenic components within cancer. This regulatory modulation assumes crucial functions governing fundamental cellular processes, including proliferation, apoptosis, EMT, invasion, migration, and metastasis.

#### NEAT1 regulates miRNAs

Numerous research inquiries have underscored NEAT1's capacity for epigenetically controlling gene expression. One illustrative example involves NEAT1's contribution to the inhibition of miR-129-5p expression, which is accomplished by increasing the DNA methylation of miR-129 (Lo et al., 2016[52]). Furthermore, the disruption of the BRCA1/NEAT1/miR-129-5p/WNT4 axis has been implicated in advancing breast tumorigenesis. Additionally, NEAT1 has exhibited the ability to modulate miR-17, although the precise mechanism driving this effect warrants further comprehensive examination (Wang et al., 2018[74]) positively.

#### MiRNAs regulate NEAT1

Recent research suggests that miRNAs directly impact lncRNAs by explicitly targeting them, reducing expression within cancer cells. Notably, miR-124-3p has been identified as a suppressor of NEAT1 expression. Reduced levels of miR-124-3p have been observed in ovarian cancer patients, suggesting its potential tumor-suppressive function through the modulation of NEAT1 expression (Chai et al., 2016[7]). Furthermore, miR-370-3p, miR-548ar-3p, miR-449a, miR-384, and miR-126 have demonstrated the capacity to significantly downregulate NEAT1, underscoring the interactions between these miRNAs and lncRNAs (Ke et al., 2016[33]; Lulli et al., 2020[54]; You et al., 2014[85]; Zeng et al., 2020[91]; Zhu et al., 2019[102]). Intriguingly, mature miRNAs might assume roles as transcriptional regulators within the nucleus. For instance, miR-140 binds to NEAT1 at the 974-998 bp region in the nucleus, thereby enhancing NEAT1 stability (Gernapudi et al., 2016[19]). The mechanisms through which miRNAs contribute to an intricately regulated NEAT1 expression warrant more in-depth investigation.

### Binding to proteins and transcription factors 

NEAT1 has garnered attention due to its capacity to interact with specific RNA-binding proteins, resulting in the assembly of functional ribonucleoprotein complexes. These complexes, termed NEAT1-associated RNPs, are characterized by RNA-binding proteins featuring prion-like domains that exhibit an inclination for aggregation. These sizable ribonucleoprotein entities, paraspeckles, represent a subset of membrane-less nuclear bodies. NEAT1 is comprised of three distinct RNA domains denoted A, B, and C, each serving specific roles in stabilization (A), isoform switching (B), and paraspeckle assembly (C). Remarkably, the central C domain encompasses smaller subdomains with a strong affinity for essential paraspeckle proteins like NONO and SFPQ, prompting subsequent polymerization along the NEAT1 RNA (Hirose et al., 2019[24]). However, additional investigations are warranted to elucidate the precise repertoire of proteins and transcription factors that engage with NEAT1 in the context of LCs. 

NEAT1 demonstrates affiliations with various transcription factors, exerting regulatory effects on their activity within specific gene promoter regions, either augmenting or repressing gene transcription (Wang et al., 2017[75]; Wen et al., 2020[77]). For instance, NEAT1's interplay with CDC5L leads to the recruitment of this factor to the promoter region of AGRN, thereby fostering prostate cancer progression (Li et al., 2018[46]). Furthermore, NEAT1 serves as a conduit between distinct proteins, wherein the interlinking of these proteins can be disrupted by RNase intervention. This phenomenon is exemplified by NEAT1's involvement in establishing a pivotal connection between SIN3A and FOXN3. While FOXN3 carries a transcription-activating domain but lacks DNA-binding capability, SIN3A possesses a DNA-binding domain without a transcription-activating motif.

Consequently, the concerted action of these proteins is vital for proper transcriptional regulation. The complex involving SIN3A, NEAT1, and FOXN3 ultimately governs downstream transcriptional events involving GATA3 and TJP1, thus influencing EMT in breast cancer (Li et al., 2017[45]). Moreover, NEAT1's interactions extend to nuclear receptors, a specialized transcription factor class that translocates from the cellular membrane to the nucleus upon ligand binding. NEAT1's interaction with the nuclear receptor ERa results in the ERa recruitment to the promoter region of AQP7, thereby promoting the development of steatosis in hepatic cancer cells (Fu et al., 2019[18]).

### Subcellular localization and nuclear paraspeckle formation 

#### Impact on RNA processing and splicing 

NEAT1 exhibits multifaceted involvement in RNA processing and splicing mechanisms. It assumes a role as a ceRNA, effectively sequestering miRNAs to modulate the expression profiles of their target genes during cancer development (Li et al., 2021[42]). Moreover, NEAT1 demonstrates regulatory influence over the alternative splicing of the lncRNA Neat1, thereby impeding the invasion and metastasis processes in NSCLC (Cong et al., 2022[10]). Furthermore, NEAT1's interaction with RNA targets is direct, constituting a mechanism for retaining RNA within paraspeckles (Jacq et al., 2021[29]). NEAT1's exosome transport expands its repertoire and allows its transfer between prostate cancer cells, which serves as a ceRNA for miR-205-5p. This action leads to the elevation of RUNX2 levels, subsequently promoting the osteogenic differentiation of human bone marrow-derived mesenchymal stem cells (hBMSCs) (Mo et al., 2021[58]).

#### Regulation of gene expression through paraspeckle-associated proteins

NEAT1 plays a pivotal role in gene expression regulation through its interactions with paraspeckle-associated proteins. NEAT1 contributes significantly to these nuclear bodies' architecture as a foundational element within paraspeckles. These specialized protein assemblies form upon the NEAT1 lncRNAs and influence cellular processes by sequestering various proteins or RNA molecules. Notably, paraspeckles are elevated in response to diverse cellular stressors and specific developmental stages. NEAT1's functionality extends further as a versatile molecular scaffold, impacting paraspeckle morphology and influencing the interaction dynamics between RNA-binding proteins and transcripts within these structures (Knutsen et al., 2022[35]; Mamontova et al., 2022;[57] Wang et al., 2018[74]).

Moreover, NEAT1 demonstrates a direct RNA-binding capability, contributing to retaining RNA within paraspeckles (Knutsen et al., 2022[35]; Spiniello et al., 2018[68]). In the context of NSCLC, NEAT1's involvement encompasses the regulation of alternative splicing within the lncRNA Neat1. This action effectively hampers the invasion and metastasis processes associated with the disease (Mamontova et al., 2022[57]). Furthermore, NEAT1 engages in the role of a ceRNA, a function involving the sequestration of miRNAs to induce alterations in the expression profiles of their downstream target genes. This intricate interplay of NEAT1 with miRNAs has significant implications for the progression of various cancers (Knutsen et al., 2022[35]; Reichelt-Wurm et al., 2022[64]).

## Clinical Implications of NEAT1 in Lung Cancer

**Figure 3:**
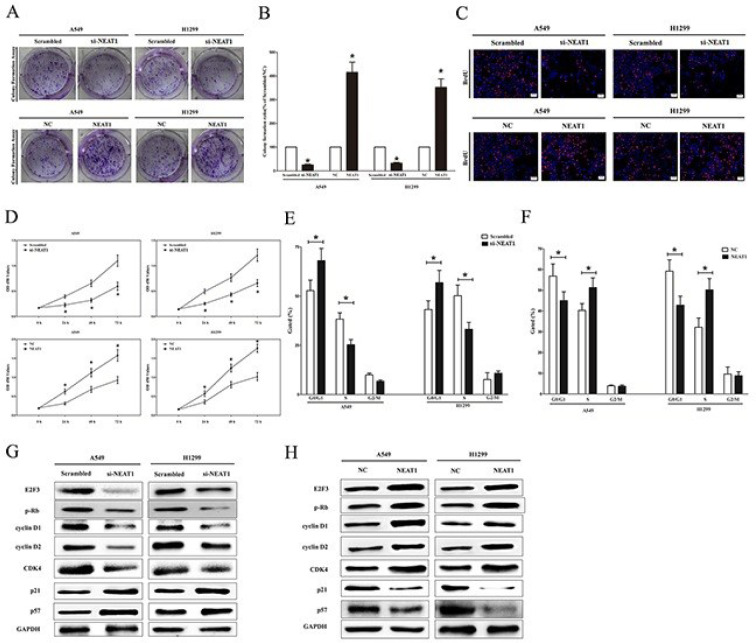
NEAT1 facilitates *in vitro* growth of NSCLC cells. In this study, the authors investigated the role of NEAT1 in promoting the growth of NSCLC cells through a series of experimental assays. (A) Representative photomicrographs captured during the colony formation assay following transfection for fourteen days are presented. (B) The statistical analysis results on the colony formation assay are illustrated. (C) After transfection, representative images displaying BrdU staining in A549 and H1299 cells are shown. The scale bar corresponds to 100 μm. (D) The outcomes of CCK8 assays on A549 and H1299 cells post-transfection are depicted. (E, F) Cell-cycle analysis was executed following transfection for forty-eight hours. Flow cytometric analysis quantified the DNA content. (G) The protein expression levels of E2F3, p-Rb, cyclin D1, cyclin D2, CDK4, p21, and p57 were assessed in transfected A549 and H1299 cells. All assays were performed in triplicate, and statistical significance was evaluated using the student's t-test. The data are presented as means ± SEM, with * indicating P < 0.05 (Sun et al., 2016).

LCs remain a pervasive malignancy, the foremost cause of cancer-related mortality globally (Jacq et al., 2021[29]). Dominating the LCs landscape is NSCLC, accounting for approximately 80 % of cases. Sun and colleagues discerned a link between elevated NEAT1 expression in NSCLC and shortened overall survival, attributing this effect to NEAT1's role in fueling cancer cell growth and metastasis (Figure 3(Fig. 3)) (Sun et al., 2016[69]). Further exploration has shed light on NEAT1's role as a ceRNA for E2F3, a crucial oncogene in advancing NSCLC. In this capacity, NEAT1 acts as a molecular sponge for hsa-miR-377-3p, thereby counteracting the inhibitory interaction between hsa-miR-377-3p and E2F3, which leads to the alleviation of E2F3 expression suppression. Moreover, NEAT1's involvement in promoting NSCLC growth, migration, and invasion has been elucidated through its interaction with miR-98-5p, miR-101-3p, and miR-376b-3p. By sequestering these miRNAs, NEAT1 enhances the expression of key mediators, including MAPK6 (Wu et al., 2019[78]), SRY-box transcription factor 9 (SOX9) (Kong et al., 2019[37]), and sulfatase 1 (SULF1) (Chen et al., 2020[8]), respectively). These targeted genes play pivotal roles in influencing the trajectory of cancer progression (Huang et al., 2019[26]; Lee et al., 2016[40]; Long et al., 2012[53]).

**Figure 4:**
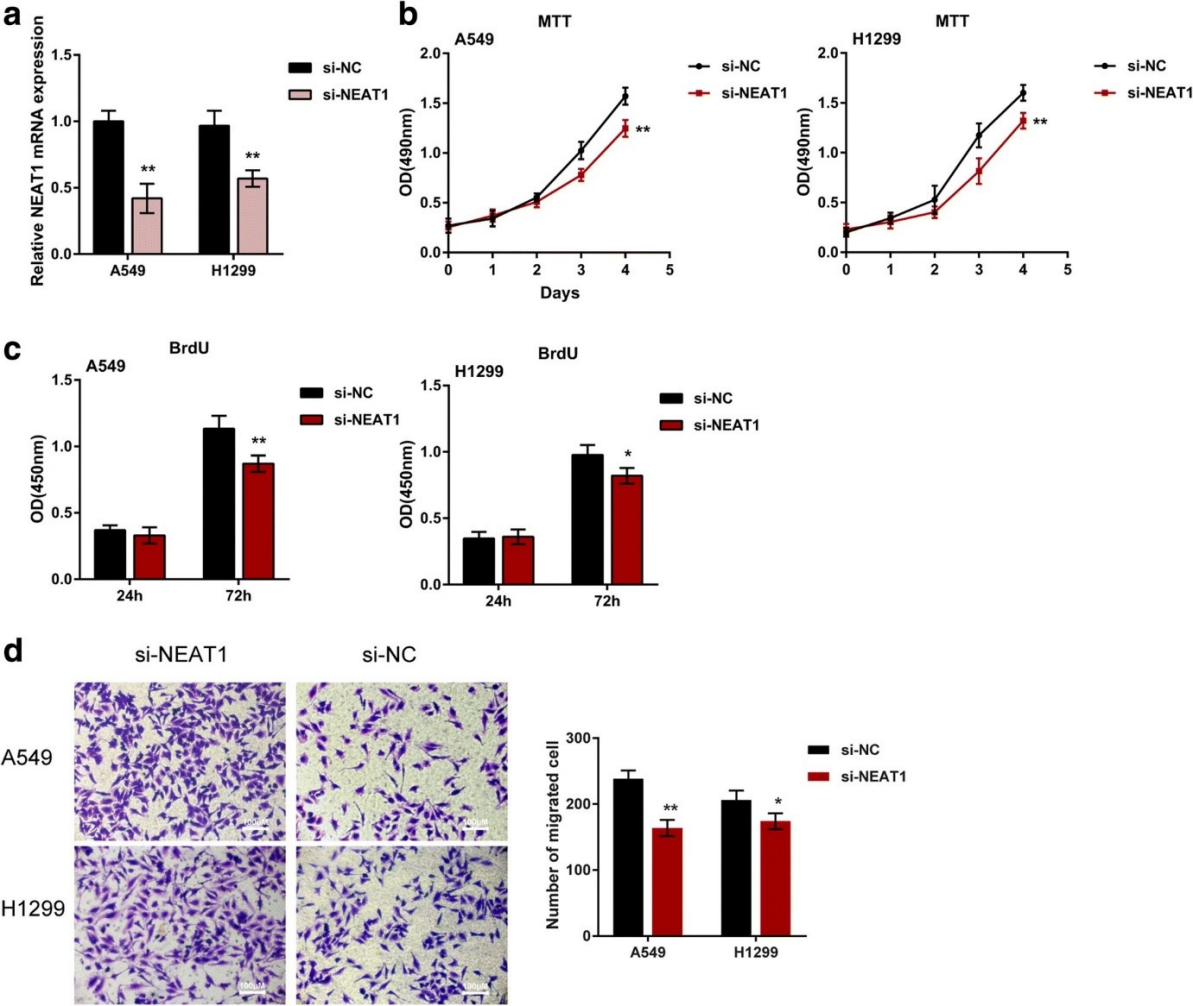
Investigation of the influence of NEAT1 on the growth and migratory behavior of lung cancer (LC) cells using the following experimental strategy: (a) Silencing of NEAT1 was achieved through si-NEAT1 transfection in A549 and H1299 cells, and the reduction in NEAT1 expression was confirmed by real-time PCR analysis. (b-c) To gauge LC cell proliferation, MTT and BrdU assays were performed. (d) The assessment of LC cell migration was carried out through Transwell assays. The presented outcomes represent the mean value and the standard deviation derived from three distinct experimental replicates. The significance of the results is indicated by *P < 0.05 and **P < 0.01 (Zhou et al., 2018).

NEAT1 exhibits heightened expression in LA, driving tumorigenesis. Through its miRNA sequestration capacity, NEAT1 fosters tumor cell proliferation (Ding et al., 2021[11]). NEAT1 has demonstrated activation of the TLR4/NF-κB pathway within LA to amplify cell proliferation. It also triggers autophagy by modulating the miR-128-3p/ADAM28 axis, suppressing apoptosis. Additionally, NEAT1 contributes to sepsis-associated encephalopathy by promoting ferroptosis via the miR-9-5p/TFRC and GOT1 pathway. Furthermore, NEAT1 is implicated in LA progression through its interaction with ATF2. However, the precise role of NEAT1 within the context of LA and its broader association with the disease remains fully elucidated (Feng et al., 2022[15]; Guo et al., 2021[22]; Zhou et al., 2018[100]). Figure 4(Fig. 4) (Reference in Figure 4: Zhou et al., 2018[100]) examined the influence of NEAT1 on the proliferation and migration of LC cells.

Overexpression of NEAT1 has been linked to adverse features in NSCLC, such as higher TNM stage, larger tumor size, lymph node metastasis, and poorer prognosis (Zhang et al., 2017[92]). NEAT1 has been identified as a facilitator of cisplatin sensitivity in NSCLC through its interaction with miR-98-5p and its influence on copper transporter 1 (CTR1) (Jiang et al., 2016[32]; Zhong et al., 2018[99]). Wu et al. further demonstrated the involvement of NEAT1/hsa-mir-98-5p/MAPK6 in the progression of NSCLC (Wu et al., 2019[78]). In the realm of miRNA modulation, NEAT1 has been shown to act as a ceRNA against miR-377-3p, impacting the expression of the transcription factor E2F3 (Sun et al., 2016[69]; Zhang et al., 2017[92]). A separate investigation uncovered NEAT1's ability to sponge miR-101-3p, consequently promoting the invasion of NSCLC by targeting the transcription factor SOX9 (Kong et al., 2019[37]; Symon and Harley, 2017[70]). The oncogenic role of NEAT1 in NSCLC progression is underscored by its involvement in the miR-101-3p/SOX9/Wnt/ß-catenin and has-miR-376b-3p/SULF1 axes (Chen et al., 2020[8]). Furthermore, NEAT1 has been shown to upregulate HMGB2, a target gene of miR-181a-5p, by competitively sponging miR-181a-5p (Li et al., 2018[44]). Collectively, these findings illuminate NEAT1's intricate involvement in the regulatory networks of NSCLC, implicating it as a potential therapeutic target for intervention.

**Figure 5:**
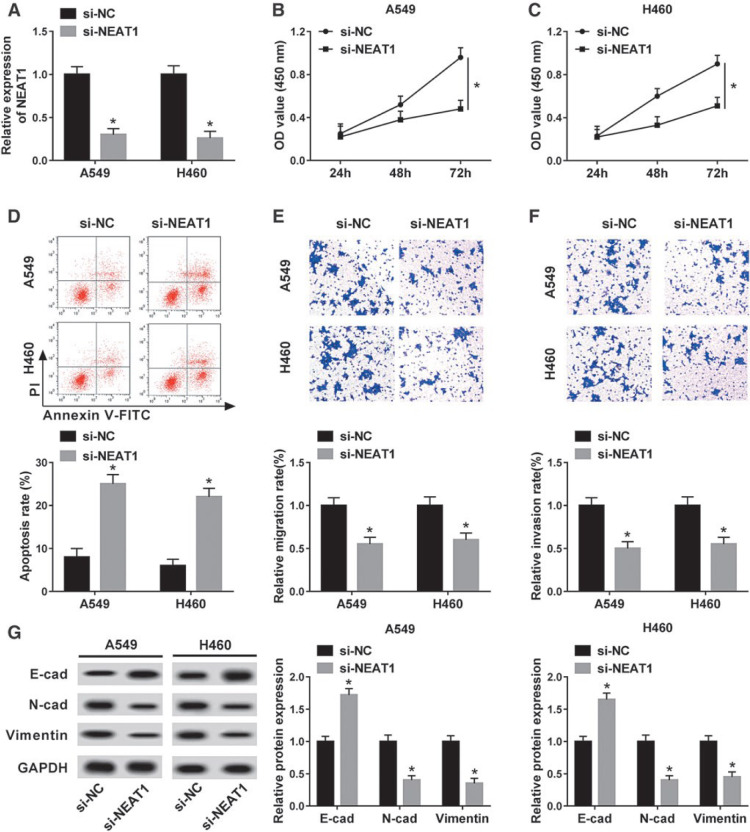
Illustrates the impact of NEAT1 knockdown on several cellular processes, including cell proliferation, migration, invasion, apoptosis, and EMT in NSCLC *in vitro*. (A) The efficacy of NEAT1 downregulation in si-NEAT1 cell lines (A549 and H460) is demonstrated by quantification through qRT-PCR. (B, C) Furthermore, the effect of si-NEAT1 on cell proliferation in A549 and H460 cells is assessed via the CCK8 assay. (D) Additionally, the influence of si-NEAT1 on cell apoptosis is evaluated using flow cytometry. (E, F) Transwell experiments are conducted to investigate the impact of si-NEAT1 on cell migration and invasion in A549 and H460 cell lines. (G) The expression levels of EMT marker proteins are analyzed through Western blotting and normalized to GAPDH. Significance levels are indicated by *p < 0.05. Abbreviations used include CCK8 (Cell Counting Kit-8), EMT, and qRT-PCR (quantitative real-time polymerase chain reaction) (Zhao et al., 2020a).

Zhao et al. indicated that NEAT1 exhibits elevated expression levels in both NSCLC tissues and cell lines. Silencing NEAT1 suppresses cell proliferation, invasion, and migration, along with the induction of apoptosis in NSCLC cell lines (Figure 5(Fig. 5), Reference in Figure 5: Zhao et al., 2020[95]). Moreover, NEAT1 is shown to interact with miR-153-3p in NSCLC directly. Further investigations revealed that the enhancement of miR-153-3p leads to a restraint on cell progression, while inhibition of miR-153-3p counteracts the inhibitory impact caused by si-NEAT1 in NSCLC cell lines. Additionally, si-NEAT1 impedes the Wnt/ß-catenin signaling pathway, which is subsequently reactivated by the miR-153-3p inhibitor (Zhao et al., 2020[95]).

## NEAT1's Implications in Cancer Therapy

NEAT1 has garnered attention for its involvement in developing and progressing several cancer types, including CRC, gallbladder, breast, prostate, NSCLC, and triple-negative breast cancer. It exerts multifaceted roles in cancer-related processes, encompassing chemoresistance, cancer stemness, and tumor immunosurveillance. In the context of CRC, NEAT1 depletion amplified the inhibitory effects of photodynamic therapy on cell viability and apoptosis (Liu et al., 2021[50]). NEAT1 exhibited an oncogenic role in gallbladder cancer by upregulating survivin and promoting cancer progression through its interaction with miRNA-335 (Yang et al., 2020[84]). Notably, NEAT1 was associated with therapy resistance in breast cancer and correlated with adverse clinical outcomes (Knutsen et al., 2022[35]). Moreover, NEAT1 demonstrated its significance in prostate cancer, contributing to aerobic glycolysis, thereby attenuating tumor immunosurveillance by T cells (Xia et al., 2022[81]). In NSCLC, NEAT1 was pivotal in regulating ferroptosis sensitivity, influencing cancer cell fate (Wu and Liu, 2021[79]). Lastly, in triple-negative breast cancer, NEAT1 modulates chemoresistance and influences cancer stemness (Shin et al., 2019[66]).

The established cancer treatment modalities encompass surgical intervention, chemotherapy, and radiotherapy. Nonetheless, a subset of cancer patients demonstrates a challenging clinical scenario characterized by metastasis and limited responsiveness to conventional chemotherapy or radiotherapy. This scenario emerges from the intricate interplay of tumor heterogeneity, the tumor microenvironment, and the malfunctioning of genes linked to therapeutic resistance (Gottesman et al., 2016[20]; Kuczynski et al., 2013[38]; Rebucci and Michiels, 2013[63]). Many studies have been conducted to explore the interrelation between NEAT1 and resistance to chemotherapy or radiotherapy across diverse cancer types. The findings have consistently unveiled that the depletion of NEAT1 can enhance the susceptibility of cancer cells to radiation or pharmacological agents by disrupting NEAT1-mediated ceRNA networks. Consequently, disrupting the feedback loop within the NEAT1/miRNA/target axis offers a promising avenue to surmount therapeutic resistance challenges in cancer treatment. 

## Future Directions

Emerging research has underscored the pivotal involvement of NEAT1 in the intricate landscape of LC development and progression. To further advance our understanding, several avenues of investigation warrant exploration. One crucial area is the intricate molecular interactions NEAT1 engages in with various molecules, including RNAs, proteins, and DNA promoter regions, that collectively contribute to the upregulation of tumorigenic factors. Unraveling these complex associations could unveil new insights into the underlying mechanisms of LC progression. Furthermore, it is imperative to delve into the mechanisms responsible for the elevation of NEAT1 in cancers, whether through transcriptional activation or alterations in stability. Additionally, the process by which NEAT1 is secreted from one cell type and subsequently delivered to cancer cells via exosomes holds potential implications for future therapeutic strategies (Gu et al., 2022[21]). Understanding these mechanisms could pave the way for innovative treatment modalities for LCs.

Lastly, the potential utility of NEAT1 as a biomarker for LC diagnosis and prognosis remains an intriguing avenue for investigation. Ascertaining its diagnostic and prognostic value in clinical settings could revolutionize patient management. Additionally, exploring the therapeutic targeting of NEAT1 as a potential avenue for LC treatment could open new doors for personalized therapies.

## Conclusion

LCs, particularly NSCLC, remain a global health challenge with high prevalence and mortality rates, necessitating novel approaches for improved outcomes. LncRNAs offer promising insights into the molecular intricacies of LCs, and NEAT1 stands out as a significant player in this landscape. Its roles extend across diverse cancer types, including NSCLC, where it influences critical cellular functions linked to tumorigenesis.

In NSCLC, NEAT1 functions as an oncogenic lncRNA, showcasing robust expression within cancer tissues. Its engagement with various molecular pathways, particularly miRNA interactions and signaling cascades, underscores its central role in promoting tumor growth and metastasis. NEAT1's involvement in regulatory networks, including its interactions with other molecules, shapes cancer cell behavior. Promisingly, NEAT1 holds diagnostic potential, correlating with advanced disease stages, lymph node metastasis, and poor prognoses. This suggests its utility as a valuable biomarker for disease prognosis and treatment guidance. NEAT1's multifaceted engagement in LC biology positions it as a potential therapeutic target and diagnostic tool. Further research is necessary to unravel its intricate mechanisms and explore its therapeutic potential. As understanding deepens, NEAT1 offers avenues for advancing LC's knowledge, potentially revolutionizing patient management.

## Declaration

### Ethics approval and consent to participate

Not applicable to a review article.

### Consent for publication 

All authors gave their consent for publication. 

### Availability of data and materials 

It is not applicable as no novel data were generated for this review article. 

### Competing interests 

No authors have any conflict of interest or competing interests to declare. 

### Funding 

No funding was received to perform this study.

### Author contributions 

MSH, OA, GG and AG researched the data. WHA, IK, SIA and ASAA wrote the first draft of the manuscript. NK, AC and SKS edited the manuscript. KD was responsible for conceptualization and supervision. All authors reviewed and approved the final version of the manuscript. Gaurav Gupta is the guarantor of this work.

### Acknowledgments 

None.
